# Functional Limb Preservation after Surgical Distal Venous Arterialization with Free Flap in Chronic Limb-Threatening Ischemia with Severe Foot Arterial Disease

**DOI:** 10.70352/scrj.cr.26-0099

**Published:** 2026-04-28

**Authors:** Chihiro Nakai, Shinsuke Kikuchi, Yuya Tamaru, Tsutomu Doita, Daichi Mizushima, Hirofumi Jinno, Kazuki Takahashi, Takayuki Uramoto, Keisuke Kamada, Seima Ohira, Naoya Kuriyama, Yuri Yoshida, Daiki Uchida, Hiroyuki Kamiya, Toshihiko Hayashi, Takanori Fujiki, Nobuyoshi Azuma

**Affiliations:** 1 Department of Vascular Surgery, Asahikawa Medical University, Asahikawa, Hokkaido, Japan; 2 Department of Cardiovascular Surgery, Osaka University Graduate School of Medicine, Suita, Osaka, Japan; 3 Department of Cardiovascular Surgery, Sapporo Kosei General Hospital, Sapporo, Hokkaido, Japan; 4 Department of Vascular Surgery, Asahikawa City Hospital, Asahikawa, Hokkaido, Japan; 5 Department of Cardiac Surgery, Asahikawa Medical University, Asahikawa, Hokkaido, Japan; 6 Department of Plastic and Reconstructive Surgery, Asahikawa Medical University, Asahikawa, Hokkaido, Japan; 7 Japanese Red Cross Shimizu Hospital, Kamikawa-gun, Hokkaido, Japan; 8 Department of Chronic Limb-Threatening Ischemia Research, Asahikawa Medical University, Asahikawa, Hokkaido, Japan

**Keywords:** surgical distal venous arterialization, free flap, chronic limb-threatening ischemia

## Abstract

**INTRODUCTION:**

In chronic limb-threatening ischemia (CLTI), especially with diabetes and dialysis dependence, below-the-ankle arterial lesions and severe calcification often limit distal bypass options. While surgical distal venous arterialization (sDVA) can restore perfusion, achieving wound healing requires additional surgical strategies such as free flap transfer to provide adequate soft tissue coverage.

**CASE PRESENTATION:**

A male patient in his 70s with diabetes and end-stage renal disease on hemodialysis presented with a right toe ulcer and rest pain. He had severe intradialytic hypotension and reduced cardiac function (ejection fraction: 22%) with multivessel coronary artery disease. Coronary artery bypass grafting (CABG) with 5 grafts was performed, and balloon angioplasty of the peroneal artery was added 53 days later to improve infrapopliteal perfusion. Cardiac function improved to an ejection fraction of 55% within 1 month, but the toe ulcer progressed to total toe gangrene. Six months later, the patient was readmitted with a deep foot infection. Despite the infection, lower limb muscle strength was preserved. sDVA was performed using a popliteal artery–posterior tibial vein bypass with the ipsilateral great saphenous vein, combined with sequential anastomosis to the diseased tarsal artery and Lisfranc-level amputation. Following sDVA, extensive debridement of infected tissue resulted in a large soft tissue defect. During the waiting period for definitive wound coverage, rheopheresis therapy using Rheocarna was administered to promote wound bed preparation. A free latissimus dorsi musculocutaneous flap was subsequently transferred to achieve wound closure. Four years after the initial intervention, the patient maintains favorable cardiac and limb function without ulcer recurrence.

**CONCLUSIONS:**

We report a case of CLTI with below-the-ankle disease in a dialysis-dependent patient, successfully treated with CABG, endovascular therapy, rheopheresis therapy, sDVA, and free flap transfer. This case underscores the importance of combining revascularization and soft tissue reconstruction to achieve wound healing and preserve ambulatory function as key goals in managing complex below-the-ankle arterial lesions.

## Abbreviations

ABI
: ankle-brachial index
CABG
: coronary artery bypass grafting
CLTI
: chronic limb-threatening ischemia
DM
: diabetes mellitus
DVA
: distal venous arterialization
ESRD
: end-stage renal disease
EVT
: endovascular therapy
GSV
: great saphenous vein
LAD
: left anterior descending artery
LITA
: left internal thoracic artery
LVEF
: left ventricular ejection fraction
sDVA
: surgical distal venous arterialization
SPP
: skin perfusion pressure

## INTRODUCTION

Treatment strategies for comprehensive CLTI are guided by the PLAN concept (Prognosis, Limb severity, and Anatomical complexity), as proposed in the Global Vascular Guidelines.^1)^ In patients with limited life expectancy, particularly those unlikely to survive beyond 2 years, open surgical bypass is generally avoided due to high perioperative risk. However, in cases complicated by DM and ESRD, high limb severity and complex arterial anatomy often render EVT insufficient, especially when below-the-ankle arterial lesions are present.^2,3)^ In such scenarios, revascularization alone may not achieve wound healing. We report a high-risk CLTI case with multivessel coronary artery disease, in which prognosis was first secured by CABG. Subsequently, a multimodal approach combining EVT, sDVA, rheopheresis therapy (Rheocarna; Kaneka, Osaka, Japan), and free flap transfer was employed to address extensive tissue loss, aiming for functional limb preservation through wound healing and maintenance of ambulatory function. Despite the initially limited prognosis, the patient achieved durable wound closure and preserved mobility, surviving for 4 years postoperatively. This case highlights the potential of a versatile, staged strategy integrating revascularization, adjunctive therapies, and soft tissue reconstruction for limb salvage in complex CLTI with below-the-ankle arterial disease.

## CASE PRESENTATION

**Fig. 1:**
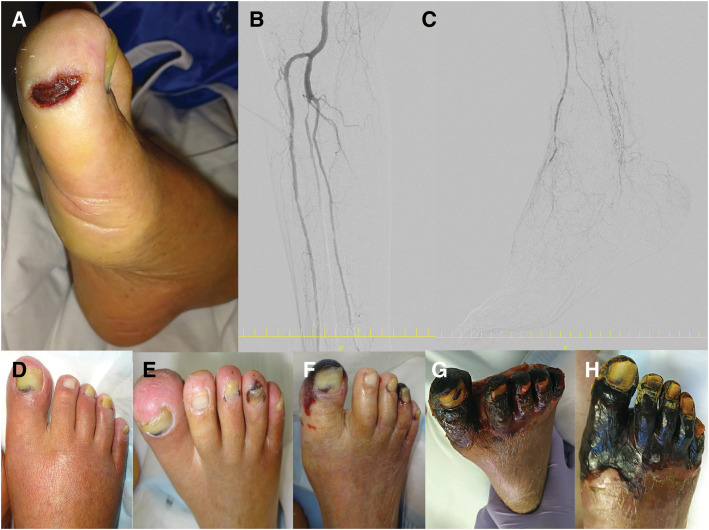
Initial examination findings, lower extremity arterial imaging, and serial changes in foot wounds after CABG. A small ulcer was noted on the right first toe, but no infection was present (**A**). Preoperative lower extremity angiography revealed no significant stenosis up to the popliteal artery. The anterior tibial artery showed segmental stenoses, the peroneal artery was occluded, and the posterior tibial artery was extensively diseased (**B**). Below the ankle, both the dorsalis pedis and plantar arteries exhibited severe disease (**C**). Serial changes in limb severity and wound progression are shown at initial presentation (**D**), POD 1 (**E**), POD 6 (**F**), POD 30 (**G**), and POD 83 (**H**; 30 days after peroneal artery balloon angioplasty). CABG, coronary artery bypass grafting

A man in his 70s with DM and ESRD on hemodialysis was referred to our department for evaluation and treatment of a right first toe ulcer (Wound grade [W] 1) with resting pain during dialysis-associated hypotension (**Fig. 1A**). On physical examination, arterial pulses were palpable at both the right groin and popliteal regions, while pedal arteries were non-palpable but detectable by Doppler US. The patient was ambulatory. ABI was 0.73 on the right side, and SPP measured 26 mmHg on the dorsum and 18 mmHg on the plantar surface of the foot, corresponding to Ischemia grade (I) 3. No signs of infection were observed (foot infection [fI] 0), and the patient was diagnosed with stage 3 CLTI (W1I3fI0).

Preoperative cardiac evaluation revealed electrocardiographic findings suggestive of ischemic heart disease. Transthoracic echocardiography showed a severely reduced LVEF of 22% with marked wall motion abnormalities in all 3 coronary territories. Coronary angiography demonstrated 99% stenosis in segments #7, #9, and #10 of the LAD; complete occlusion of segment #13 and 99% stenosis of segment #14 in the left circumflex artery; and complete occlusion from segment #2 of the right coronary artery. Lower limb arterial imaging revealed no significant lesions up to the popliteal artery. However, all 3 infrapopliteal arteries were diseased, and no identifiable main trunks of the dorsalis pedis or plantar arteries were observed (**Fig. 1B** and **1C**), making both endovascular and bypass revascularization anatomically challenging.

Given the critical coronary anatomy and poor cardiac function, revascularization of the coronary arteries was prioritized to secure the patient's prognosis. CABG was performed using the LITA to the LAD, and saphenous vein grafts to the diagonal branch, obtuse marginal branches, and the posterior descending branch of the right coronary artery. The total operative time was 3 h and 20 min. Postoperatively, the patient required mechanical circulatory support with an Impella 5.0 (Abiomed, Danvers, MA, USA) for 5 days and intensive care for approximately 1 month. During this perioperative period, the small toe ulcer progressed to total gangrene of all toes due to worsening peripheral ischemia. Fifty-three days after CABG, balloon angioplasty of the peroneal artery was performed to improve infrapopliteal perfusion. Although a temporary improvement in flow was achieved, the forefoot ischemia continued to progress, ultimately resulting in total toe gangrene (**Fig. 1D**–**1H**).

Six months after transfer to a rehabilitation facility, the patient developed a foot infection and was readmitted to our department. At that time, the limb severity had progressed to extensive necrosis (W3), severe ischemia (I3), and deep tissue infection (fI2), corresponding to WIfI stage 4, the most severe stage of CLTI. The patient’s nutritional status was preserved, with a serum albumin level of 3.4 g/dL. Prognostic evaluation using the Japanese CLTI registry-based risk calculator estimated a 30-day survival rate of over 95% and a 2-year survival rate of 79% with surgical revascularization (compared to 58% with EVT), classifying the patient as standard risk.^4)^ These findings supported the feasibility of surgical intervention.

Given the absence of graftable distal arteries in the foot (**Fig. 1B**), we planned a 2-pronged surgical strategy. First, sDVA was selected for revascularization via the posterior tibial vein. However, a major limitation of sDVA in this case was the extensive tissue loss, which compromised the availability of venous outflow pathways. Therefore, as a second measure, we planned an additional bypass to the diseased tarsal artery, which, despite its pathology, demonstrated Doppler-detectable flow on US. This was intended to supplement the limited perfusion expected from sDVA and potentially preserve some arterial inflow. As an exit strategy, we aimed to achieve functional limb preservation through the creation of a new runoff and soft tissue coverage using a free flap based on the sDVA inflow, as previously reported.^5)^

**Fig. 2:**
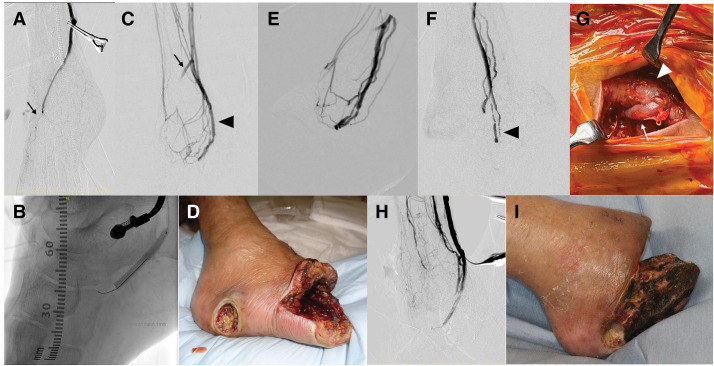
Procedure for tarsal artery reconstruction and sDVA. The surplus graft was first anastomosed end-to-side to the main DVA graft and then used to reconstruct the tarsal artery (arrow, **A**). A 4-Fr sheath was inserted retrogradely into the PTV, and the valves of the PTV and plantar vein were disrupted using a 4.0-mm peripheral balloon (**B**). The GSV harvested from the ipsilateral thigh was anastomosed end-to-side to the PTV as a single vein graft, and the proximal PTV was ligated to complete the sDVA (arrowhead). The graft to the tarsal artery in panel (**C**) remained patent but with limited flow (arrow). Forefoot debridement was performed and managed with open drainage following sDVA (**D**). At 1-month post-sDVA, graft angiography demonstrated good DVA flow (**E**), whereas at 2 months, severe stenosis of the plantar vein was observed, resulting in DVA occlusion (arrowhead, **F**). Redo sDVA was performed by anastomosing another PTV (arrow), which had undergone valve destruction, to the previous sDVA graft (arrowheads) using an end-to-side anastomosis (**G**). Intraoperative angiography during redo sDVA for graft occlusion is shown in (**H**), and postoperative foot findings after debridement following redo sDVA are shown in (**I**). DVA, distal venous arterialization; GSV, great saphenous vein; PTV, posterior tibial vein; sDVA, surgical distal venous arterialization

The planned sDVA with diseased-artery reconstruction procedure was performed under lower extremity nerve blockade with sedation to minimize perioperative stress.^6)^ The ipsilateral GSV was harvested and used in a non-reversed fashion after valve destruction with a valvulotome. The below-the-knee popliteal artery was selected as the inflow for sDVA, and the posterior tibial vein at the ankle level served as the outflow. A surplus segment of the GSV graft was anastomosed to the tarsal artery, whose patency had been confirmed preoperatively by US, and its proximal end was connected end-to-side to the main sDVA graft (**Fig. 2A**).

As previously described,^7)^ a sheath was inserted retrogradely into the posterior tibial vein, and a guidewire was advanced across the plantar venous arch. Multiple venous valves were disrupted using a balloon catheter to establish retrograde venous perfusion. After ligation of the proximal posterior tibial vein, the GSV graft was anastomosed end-to-side to the vein (**Fig. 2B**). Completion angiography demonstrated slow but adequate flow, with antegrade perfusion into the tarsal artery confirmed (**Fig. 2C**). Following revascularization, the infected forefoot was extensively debrided and managed with an open Lisfranc-level amputation and drainage (**Fig. 2D**), creating the wound bed for subsequent reconstruction.

Although DVA perfusion was favorable 1 month postoperatively (**Fig. 2E**), the patient contracted COVID-19, delaying further treatment. By the second postoperative month, significant graft stenosis developed (**Fig. 2F**), necessitating a revision of sDVA. A parallel posterior tibial vein was selected, and balloon valvulotomy was repeated to establish a new DVA conduit using a revised saphenous vein graft (**Fig. 2G** and **2H**). During the reintervention period, the tarsal artery graft became occluded, resulting in limited arterial inflow. The tissue defect further enlarged (**Fig. 2I**). To improve microcirculation in the residual tissue, rheopheresis therapy using Rheocarna was administered a total of 16 times, resulting in a reduction of fibrinogen levels from 351 to 196 mg/dL.

**Fig. 3:**
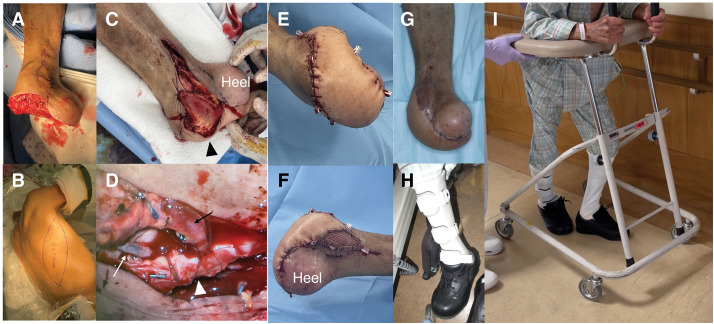
Intraoperative findings and postoperative course of flap reconstruction. Following debridement of necrotic tissue, only the calcaneal region of the foot remained intact (**A**). A latissimus dorsi musculocutaneous flap was harvested (**B**) and used to fill the soft tissue defect of the foot (**C**). The flap’s arterial inflow (black arrow) was anastomosed end-to-side to the DVA graft (arrowhead), and the flap’s venous outflow (white arrow) was anastomosed end-to-end to the posterior tibial vein to establish flap perfusion (**D**). Immediate postoperative views of the foot following flap placement are shown in (**E**) and (**F**). At 1 month postoperatively, the flap remained viable and well-integrated (**G**). A custom orthotic device was fabricated to enable forefoot weight-bearing during ambulation (**H**), and gait training was initiated 1 month after surgery (**I**). DVA, distal venous arterialization

Thereafter, a free latissimus dorsi musculocutaneous flap was transferred under general anesthesia. As only the heel could be preserved (**Fig. 3A** and **3B**), the flap was inset anteriorly to cover the forefoot defect (**Fig. 3C**). The flap artery was anastomosed end-to-side to the DVA graft, and the flap vein was anastomosed end-to-end to the posterior tibial vein, establishing reliable flap perfusion (**Fig. 3D**–**3F**). Complete wound healing was achieved by 1 month postoperatively (**Fig. 3G**). To support forefoot weight-bearing and maintain ambulatory function, a custom prosthetic device was fabricated (**Fig. 3H**). Gait training with the device was initiated (**Fig. 3I**), and the patient was subsequently transferred to a rehabilitation facility before being discharged home.

**Fig. 4:**
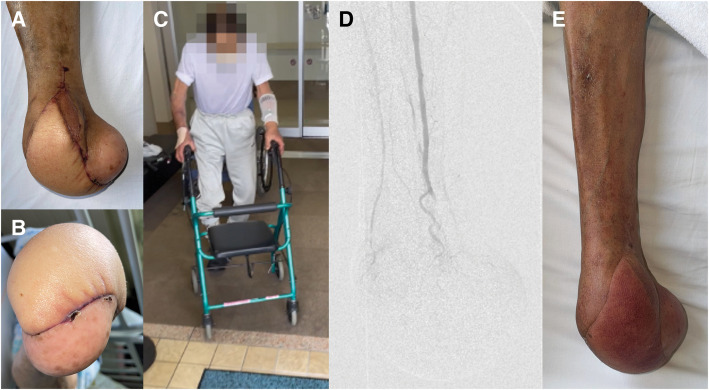
Long-term foot condition, ambulatory function, and sDVA course after surgery. At 1 year after sDVA, the foot remained intact with stable coverage (**A**, **B**). At 2 years postoperatively, the patient maintained ambulatory function using a walker (**C**). Angiographic evaluation at 2 years revealed DVA occlusion; however, the flap’s arterial inflow continued to serve as a runoff pathway, maintaining graft perfusion (**D**). At 4 years post-sDVA, the foot remained intact without recurrence of tissue loss (**E**). DVA, distal venous arterialization; sDVA, surgical distal venous arterialization

Throughout the 4 years following surgery, the patient has remained free of ulcer recurrence, with stable cardiac function (**Fig. 4A**–**4C**). Although venous perfusion through the DVA graft has ceased (**Fig. 4D**), the flap itself has become the new runoff of the vein graft, maintaining primary patency and supporting durable limb preservation (**Fig. 4E**).

## DISCUSSION

CLTI with severe foot arterial disease and dialysis dependence represents one of the most challenging clinical scenarios in vascular surgery.^3)^ Among CLTI patients, approximately 10% require concomitant open cardiac surgery, and those classified as WIfI stage 3 or 4 face particularly poor prognoses, with 2-year survival rates reported as low as 30%.^8)^ Preoperative hypoalbuminemia and foot infection are known predictors of postoperative complications. In our case, the patient’s preserved nutritional status and absence of foot infection at the time of CABG likely contributed to a stable postoperative course, enabling subsequent limb salvage interventions.

In patients with “no-option” CLTI, those lacking graftable distal arteries, sDVA has emerged as a potential revascularization strategy. However, the current evidence base remains limited. Meta-analyses report 1-year limb salvage rates of 70%–75%, with primary patency rates ranging from 44% to 71% and secondary patency around 46%.^9)^ These modest patency outcomes reflect the technical challenges of sDVA, particularly the need for effective venous valve destruction to establish retrograde perfusion. Balloon valvulotomy is commonly employed, but early restenosis at valve sites is a known limitation,^7)^ often occurring within the first postoperative month and necessitating reintervention, as seen in our case. Recent findings, including a 2025 case, have highlighted that long-term sDVA patency is influenced not only by the completeness of valve disruption but also by the intrinsic diameter and quality of the recipient vein.^7)^ These anatomical factors should be carefully evaluated during surgical planning, as they may affect the durability of venous perfusion.

Importantly, solitary DVA is often insufficient for wound healing in patients with extensive tissue loss and inframalleolar arterial disease, particularly those classified as the inframalleolar pedal disease descriptor grade 2 (IMPD-2).^10)^ In such cases, sDVA has to be integrated into a broader “exit strategy” tailored to the wound severity and arterial anatomy. When sDVA is used as the inflow for a bypass to a diseased distal artery, the entire construct becomes dependent on the patency of the DVA. If the DVA graft occludes, the downstream arterial bypass may also thrombose, leading to recurrent ischemia. Sasajima et al. proposed a combined approach using DVA as the inflow for a free flap in patients with extensive tissue loss.^5)^ Their study demonstrated that while sDVA may provide early perfusion, the transferred flap can develop into a new arterial runoff, maintaining tissue viability even after sDVA occlusion. This concept was central to our strategy: we combined sDVA with a free latissimus dorsi musculocutaneous flap and a supplemental bypass to the tarsal artery. This multimodal approach provided both immediate perfusion and long-term tissue support. At 4 years postoperatively, the patient remains ulcer-free with preserved ambulatory function, despite the eventual loss of venous perfusion through the sDVA graft, as shown in **Fig. 4D**.

This case underscores the importance of a staged, multidisciplinary approach in managing complex CLTI with severe foot arterial disease. sDVA, when combined with soft tissue reconstruction and tailored to the patient’s anatomical and systemic condition, can offer a viable path to functional limb preservation. Future studies should aim to refine patient selection, optimize technical protocols, and define long-term outcomes for sDVA-based strategies, particularly in patients with IMPD-2 disease and extensive tissue loss.

## CONCLUSIONS

We report a case of CLTI with below-the-ankle disease in a dialysis-dependent patient successfully treated with CABG, EVT, DVA, and free flap transfer. This case underscores the importance of combining revascularization and soft tissue reconstruction to achieve wound healing and preserve ambulatory function as key goals in managing complex below-the-ankle arterial lesions.

## DECLARATIONS

### Availability of data and materials

The data used for this case report are available from the corresponding author upon request.

## References

[1] Conte MS, Bradbury AW, Kolh P, et al. Global vascular guidelines on the management of chronic limb-threatening ischemia. J Vasc Surg 2019; 69: 3S–125S.e40. 31159978 10.1016/j.jvs.2019.02.016 PMC8365864

[2] Iwata S, Mizuguchi Y, Suzuki R, et al. Impact of hemodialysis duration on arterial characteristics and patient outcomes following endovascular therapy for inframalleolar occlusive disease: results from the MAVERICK study. J Vasc Interv Radiol 2025; 36: 1436–49.e5. 40467023 10.1016/j.jvir.2025.05.028

[3] Kikuchi S, Sasajima T, Inaba M, et al. Evaluation of paramalleolar and inframalleolar bypasses in dialysis- and nondialysis-dependent patients with critical limb ischemia. J Vasc Surg 2018; 67: 826–37. 28965798 10.1016/j.jvs.2017.07.116

[4] Azuma N, Takahara M, Kodama A, et al. Predictive model for mortality risk including the wound, ischemia, foot infection classification in patients undergoing revascularization for critical limb ischemia. Circ Cardiovasc Interv 2019; 12: e008015. 31771341 10.1161/CIRCINTERVENTIONS.119.008015

[5] Sasajima T, Azuma N, Uchida H, et al. Combined distal venous arterialization and free flap for patients with extensive tissue loss. Ann Vasc Surg 2010; 24: 373–81. 19765948 10.1016/j.avsg.2009.07.001

[6] Kikuchi S, Yamaguchi T, Miyake K, et al. Effectiveness and safety of ultrasound guided lower extremity nerve blockade in infragenicular bypass grafting for high risk patients with chronic limb threatening ischaemia. Eur J Vasc Endovasc Surg 2019; 58: 206–13. 31272780 10.1016/j.ejvs.2019.03.023

[7] Yoshida Y, Kikuchi S, Mizushima D, et al. Combined arterial reconstruction and surgical distal venous arterialization for limb salvage in thromboangiitis obliterans: a case report. Surg Case Rep 2025; 11: 25–0342. 10.70352/scrj.cr.25-0342 PMC12313424 40755905

[8] Wakabayashi N, Kikuchi S, Kuriyama N, et al. The impact of chronic limb-threatening ischemia on cardiac surgery. Front Surg 2022; 9: 892309. 35574536 10.3389/fsurg.2022.892309 PMC9096659

[9] Schreve MA, Vos CG, Vahl AC, et al. Venous arterialisation for salvage of critically ischaemic limbs: a systematic review and meta-analysis. Eur J Vasc Endovasc Surg 2017; 53: 387–402. 28027892 10.1016/j.ejvs.2016.11.007

[10] Houlind K, Christensen J, Hallenberg C, et al. Early results from an angiosome-directed open surgical technique for venous arterialization in patients with critical lower limb ischemia. Diabet Foot Ankle 2013: 4. 10.3402/dfa.v4i0.22713 PMC3867748 24358432

